# Structure Based Docking and Molecular Dynamic Studies of *Plasmodial* Cysteine Proteases against a South African Natural Compound and its Analogs

**DOI:** 10.1038/srep23690

**Published:** 2016-03-31

**Authors:** Thommas M. Musyoka, Aquillah M. Kanzi, Kevin A. Lobb, Özlem Tastan Bishop

**Affiliations:** 1 Research Unit in Bioinformatics (RUBi), Department of Biochemistry and Microbiology, Rhodes University, Grahamstown, South Africa; 2 Department of Genetics, Forestry and Agricultural Biotechnology Institute (FABI), Faculty of Natural and Agricultural Sciences, University of Pretoria, Pretoria, South Africa; 3 Department of Chemistry, Rhodes University, Grahamstown, South Africa

## Abstract

Identification of potential drug targets as well as development of novel antimalarial chemotherapies with unique mode of actions due to drug resistance by *Plasmodium* parasites are inevitable. Falcipains (falcipain-2 and falcipain-3) of *Plasmodium falciparum*, which catalyse the haemoglobin degradation process, are validated drug targets. Previous attempts to develop peptide based drugs against these enzymes have been futile due to the poor pharmacological profiles and susceptibility to degradation by host enzymes. This study aimed to identify potential non-peptide inhibitors against falcipains and their homologs from other *Plasmodium* species. Structure based virtual docking approach was used to screen a small non-peptidic library of natural compounds from South Africa against 11 proteins. A potential hit, 5α-Pregna-1,20-dien-3-one (5PGA), with inhibitory activity against *plasmodial* proteases and selectivity on human cathepsins was identified. A 3D similarity search on the ZINC database using 5PGA identified five potential hits based on their docking energies. The key interacting residues of proteins with compounds were identified via molecular dynamics and free binding energy calculations. Overall, this study provides a basis for further chemical design for more effective derivatives of these compounds. Interestingly, as these compounds have cholesterol-like nuclei, they and their derivatives might be well tolerated in humans.

*Plasmodium* parasites have an unmatched track record of gaining resistance to virtually all available drugs developed against them^1^. Over time, these parasites have acquired intricate strategies through which they continue to exercise their stubborn nature as colonists of their hosts^2^,^3^. Currently, the first-line malaria treatments comprise five major artemisinin based combination therapies (ACTs) as guided by World Health Organization (WHO)^4^. Over the last decade, global mortality and morbidity levels of malaria have decreased substantially with an estimated annual death rate of 0.5 million fatalities as of 2014^5^. This milestone realization is attributed to the availability of ACTs coupled with the use of insecticide treated mosquito nets (ITNs)^6^,^7^. However, ACTs could become ineffective in the near future considering that the rise and spread of artemisinin resistance in *Plasmodium falciparum* (*Pf*) has already been reported in several places in Asia^1^,^8^. This looming crisis threatens ongoing global malaria elimination strategies and the gains attained so far. To avoid similar catastrophic effects as witnessed in the case of the build-up of resistance by *plasmodia* against chloroquine in the 1980s and subsequently also by fansidar, the search for new drugs and drug targets remains a top priority. Moreover, the majority of available antimalarial drugs have toxic effects on humans hence the need for novel antimalarial drugs with exclusive toxicity against *Plasmodium* parasites is of paramount clinical importance. In terms of vaccination, an ideal malaria vaccine has remained elusive over time^9^. Recently, Mosquirix™ was approved by the European Medicines Agency (EMA) to help in the fight against malaria^10^,^11^. However, based on its protective efficacy and target group, chemotherapy still remains the leading option for the treatment of malaria infections. Deciphering the complex biochemical pathways utilized by the *Plasmodium* parasites offers an array of macromolecular structures that can be targeted for antimalarial drug development^12^,^13^,^14^. Metabolic pathways unique to the parasites, mainly haemoglobin degradation and subsequent detoxification of the heme group, nucleic acid metabolism, oxidative stress and fatty acid biosynthesis, have been of major interest for the identification of potential inhibitors.

As part of an effort to identify potential antimalarial hit compounds, our focus is on the haemoglobin degradation pathway, the most integral process for the growth and replication of *Plasmodium* parasites within the host’s erythrocytes. Through a highly ordered cascade of reactions catalysed by a group of proteases (falcipains, plasmepsins and aspartic proteases), *plasmodia* break the α- and β-globin chains of the host haemoglobin into constituent amino acids^15^,^16^,^17^,^18^. This process plays both anabolic and non-anabolic functions; a source of essential amino acids as parasites lack a *de novo* amino acid biosynthesis pathway as well as source of energy, the regulation of osmotic pressure and the creation of space in the host cell for the growing parasites. This research concentrates on falcipain (FP) proteins, namely FP-1, FP-2, FP-2’ and FP-3, found in *Pf*^19^. FPs have been implicated not only in the haemoglobin degradation process but in the erythrocyte egression and subsequent rupturing process^14^,^19^. So far, FP-2 and FP-3 have been validated as drug targets^20^ but no drug has been approved against them.

Considering the importance of natural products in drug discovery^21^,^22^, our aim was to identify potential non-peptidic hits from South African (SA) natural compounds with inhibitory potency against FP-2, FP-3 and homologs from other *Plasmodium* species. These homologs included vivapain 2 and 3 (VP-2 and VP-3) of *P. vivax*, knowlesipain 2 and 3 (KP-2 and KP-3) of *P. knowlesi*, bergheipain 2 (BP-2) of *P. berghei*, chabaudipain 2 (CP-2) of *P. chabaudi* and yoelipain 2 (YP-2) of *P. yoelii*. Additionally, we aim to determine the selectivity of identified hits on human cathepsins (Cat K and Cat L), the host homologs. Using *in silico* structure-based virtual screening (SBVS) approach, a potential hit, 5α-Pregna-1,20-dien-3-one (5PGA), was identified from a library of 23 SA natural compounds. To increase the chemical search space and the probability of obtaining more potent 5PGA like compounds, the ZINC database^23^,^24^ was searched, and 186 analogous compounds were identified. A filter based on docking energy identified five potential hits with better inhibitory potency profiles against *plasmodial* cysteine proteases, and further analysed by molecular dynamics (MD) and binding free energy calculations. Interestingly, all the potential hit compounds identified in this study showed distinct inhibitory effect against malarial proteins. Hence, they provide a starting point for further design of more effective derivatives.

## Methods

**Figure 1:**
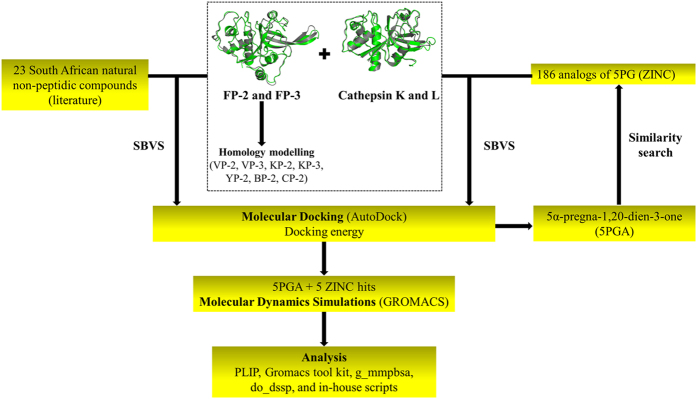
Graphical representation of the different approaches used in this study.

Figure 1 summarizes the workflow of the methodology used in this study as detailed below. The numbering of residues is based on the catalytic domain of respective proteins. For actual numbering, see Table 1.

### Protein structure data and preparation

The crystallographic structure files for FPs (FP-2 [2OUL]^25^ and FP-3 [3BWK]^26^), human cathepsins (Cat K [3OVZ]^27^ and Cat L [3OF8]^28^) were retrieved from the Protein Data Bank (PDB)^29^. High quality homology models of *P. vivax* VP-2 and VP-3, *P. knowlesi* KP-2 and KP-3, *P. berghei* BP-2, *P. chabaudi* CP-2 and *P. yoelii* YP-2 were calculated using MODELLER version 9.10^30^ as described in our earlier work^31^. Prior to docking, all crystallographic water molecules and bound ligands were removed on all 3D structures obtained from PDB.

### Hit identification from South African natural compounds

Initially, a small subset of 23 non-peptidic natural compounds (Supplementary Fig. S1) from South Africa were identified from the literature^32^,^33^ for structure based docking. These compounds have since then been entered into the South African Natural Compounds Database (SANCDB)^34^. It was not a prerequisite that the selected compounds had antimalarial activity tested before. Using Discovery Studio (DS) version 3.5 (Accelrys Software Inc. San Diego), compounds were sketched and converted to 3D structures.

### Molecular docking

All the 23 SA natural compounds were docked into all 11 proteins (nine *plasmodial* proteases and two human cathepsins) using AutoDock4.2^35^. The partial charges of the ligands were assigned using the Gasteiger-Huckel method in AutoDock tools (ADT). Docking studies were performed as detailed by Musyoka *et al.*^31^. From the docking results, a compound, 5α-Pregna-1,20-dien-3-one (5PGA), was identified as a potential hit.

### ZINC database similarity search

**Figure 2:**
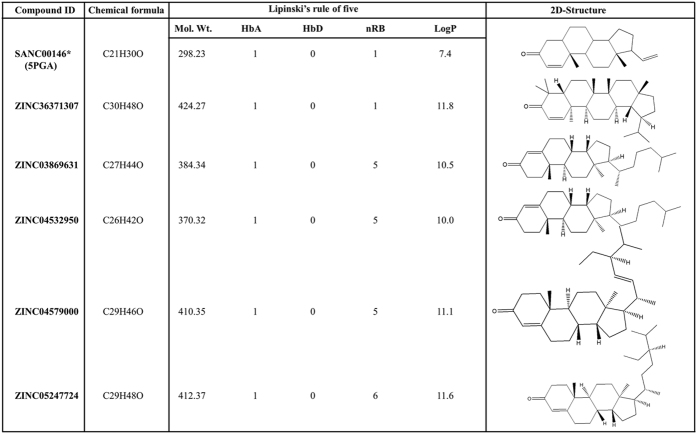
The drug-like properties, molecular weight (Mol. Wt.), hydrogen bond acceptors (HbA), hydrogen bond donors (HbD), number of rotational bonds (nRB), partition coefficient (LogP) and the 2-Dimensional (2D) structures of all compounds used in this study. Marked with asterisk is the South African hit used for structure similarity search on the ZINC database.

186 compounds analogous to 5PGA were obtained from the ZINC database and docking studies was performed as with the SA compounds. Based on the docking energy results, five hits (ZINC36371307, ZINC03869631, ZINC04532950, ZINC04579000 and ZINC052477224) were selected for further analysis. To determine the drug-likeness of the identified hits, the Lipinski’s rule of five (Ro5) were calculated using DruLiTo^36^. The interactions between ligand atoms and protein residues were determined using protein ligand profiler (PLIP)^37^. The best docked poses (lowest energy conformations) for PGA and the five selected ZINC hits in complex with all the proteins were used as the starting structures for molecular dynamics (MD). Figure 2 shows the 2D chemical structures and drug-likeness properties of 5PGA and the five hits.

### Molecular dynamics

To explore the stability and conformational flexibility (global and local) of all the protein and ligand systems under study, all-atom MD simulations were performed as described previously by Musyoka *et al.*^31^. As the force filed parameters for all ligands studied were lacking in the AMBER96 force field used for MD simulations, AnteChamber Python Parser interface (ACPYPE)^38^ was used to parametrize the required topologies, atomic types and charges. Using an *ad hoc* Python script, the generated GROMACS compatible files for the proteins and the ligands were then merged, solvated, minimized and equilibrated. Production runs of 20 ns with an integration time step of 0.2 ps were performed at a constant temperature and pressure using the leapfrog algorithm. LINCS algorithm^39^ was used to constrain all bonds during the equilibration while the particle-mesh Ewald algorithm^40^ approximated long range ionic interactions. Trajectory snapshots were stored at every 0.2 ps during the simulation period, and 3D coordinate files harvested after every 2 ns for post-dynamic analysis.

### Trajectory analysis

GROMACS analysis toolkit utilities were used to analyse MD trajectories produced during the last 12 ns of the production run to determine root mean square fluctuations (RMSF), root mean square deviation (RSMD), radius of gyration (Rg) and hydrogen bond distribution for each system. During the simulations, 3D coordinate snapshots were extracted at 2 ns interval, and PLIP was used to analyse the various interactions for each protein-ligand system. For quality assurance, the convergence of thermodynamic parameters in all systems was determined beforehand. PyMOL (The PyMOL Molecular Graphics System, Version 1.6.0.0 Schrodinger, LLC.) was used for structural alignments and visualizations. For plotting graphs, MS Excel (2013), R statistical package and Xmgrace (Grace 5.1.21) were used. To determine the evolution of the secondary structural elements in the proteins during MD simulations, the do_dssp program was used in GROMACS 4.6.5. Number of distinct hydrogen bonds formed between specific amino acids residues and ligand atoms was determined utilizing the g_hbond with the donor-acceptor set at a maximum of 0.35 nm.

### Interaction energy estimation using MM-PBSA approach

The binding energy of each protease-ligand complex (5PGA and ZINC analogues) was determined using the g_mmpbsa tool^41^. Using 6,000 snapshots structures extracted from the last 12 ns time period, the set of equations below was used to determine BFE (ΔG_*bind*_).


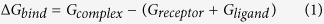



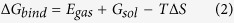



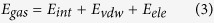



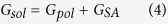



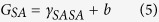


Absolute free energies of the protein-complex, apoprotein and ligands are denoted by *G*_*complex*_, *G*_*receptor*_ and *G*_*ligand*_ respectively. The Δ*G*_*bind*_ was decomposed to its individual contributions (equations 2, 3, 4, 5); a gas-phase energy (*E*_*gas*_) which is a sum of bonded (*E*_*int*_) and nonbonded terms (*E*_*vdw*_ and *E*_*ele*_); the solvation free energy (*G*_*sol*_) was decomposed into polar (*G*_*pol*_) and nonpolar (*G*_*SA*_) solvation energy components, and an entropy term (*T*Δ*S*). Polar solvation energies were determined by solving the Poisson-Boltzmann linear equation while nonpolar solvation through the solvent accessible surface area with an offset value (b) of 3.84928 kJ.mol^−1^ and surface tension proportionality (γ) set at 0.0226778 kJ.mol^−1^.Å^−2^. The individual contributions of protein residues to the three energetic components were determined through per-residue decomposition.

### System specifications

All final production MD simulations and BFE calculations were performed at the Centre for High Performance Computing (CHPC), Cape Town, South Africa. Docking studies, structure minimization, ensemble equilibration and trajectory analysis were implemented on local clusters.

## Results and Discussion

### Docking studies

Previous experiments targeting FPs using peptide based inhibitors have failed due to their poor pharmacological profiles besides being prone to degradation by host enzymes. Thus, we opted to test a small subset of SA non-peptide compounds^32^,^33^, mostly from marine sources. The compounds are mainly alkaloids and terpenes. Some of these compounds have known biological activities. For instance, 6β,7α-Diacetoxylabda-8,13-dien-15-ol (SANCDB id: SANC00228) has anticancer activity^42^, while halistanol disulfate B (SANC00619) is identified as endothelium converting enzyme inhibitor^43^.

**Figure 3:**
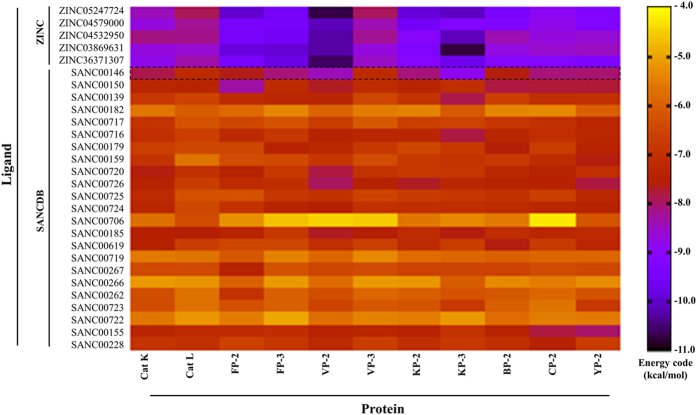
AutoDock binding energies. A heatmap showing the interaction energies of the SA subset of natural compounds and selected ZINC hits when docked against human cathepsins and *plasmodial* cysteine proteases. Shown by the dotted box are the energy profiles of the interaction between 5PGA and the corresponding protease. The energy code shows regions with interaction energy ranging from low (yellow) to high (black).

Docking results indicated that 22 out of 23 of these compounds exhibited poor binding affinities to all the proteases used (Fig. 3). In most cases, most of these poor binders had a long carbon chain or a circularised nucleus hence could not fit in the “trench-like” binding pocket, the characteristic of the cysteine proteases (Supplementary Fig. S1). Nonetheless, a small pregnadiene sterol from *Capnella thyrsoidea*, 5PGA (SANC00146), was identified as a potential hit based on its stronger binding affinity against most *plasmodial* proteases (Fig. 3). It belongs to the xenicane diterpine class of compounds. Several studies have shown the pharmacological importance of xenicane diterpines and pregnadiene derivatives; anti-tumor, antibacterial, antifungal and corticosteroids^44^. 5PGA has not been tested for antimalarial activity before. However, in a different context, it was shown to elicit an inflammatory response through release of superoxide ions in neutrophils from rabbit cells^32^.

**Figure 4:**
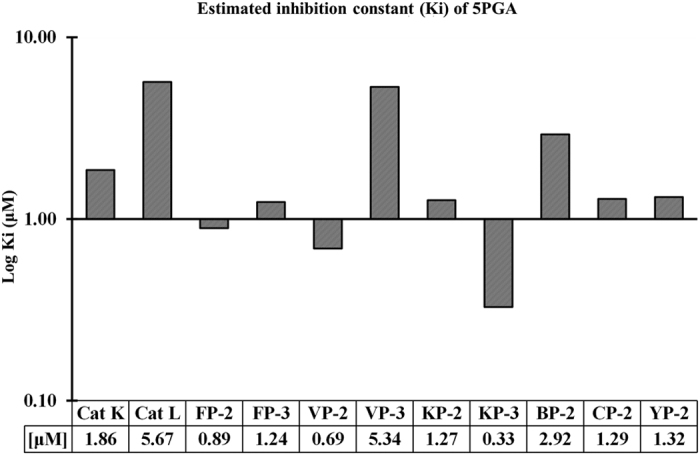
5PGA-protein predicted inhibitor constants as determined by AutoDock software.

**Figure 5:**
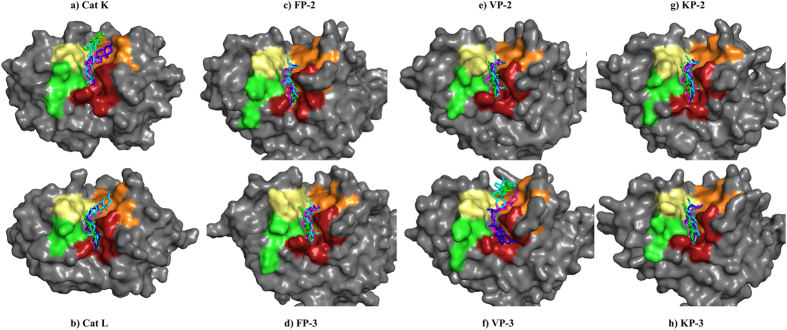
Binding poses of 5PGA (green), ZINC03869631 (magenta), ZINC04532950 (blue) and ZINC05247724 (cyan) in relation to the various subsites of cysteine proteases. S1 is shown in pale yellow, S2 in brick red, S3 in green while S1’ in orange.

5PGA had predictive inhibitory constants of up to nanomolar levels in FP-2, VP-2 and KP-3 (Fig. 4). The compound fitted perfectly to the extended S2 subsite of all *plasmodial* proteases except in VP-3 (Fig. 5). As shown in previous work^31^, amino acid residues lining S2 are key players in the binding of ligands besides conferring selectivity to the proteases. Either the carbonyl oxygen or the terminal alkene chain group of 5PGA interacted with the deepest residue in S2 in FP-2, VP-2 and CP-2 through a hydrogen bond, hence the observed stronger binding affinities. In VP-3 and Cat K, 5PGA docked in the S1’ subsite only. However, due to its small size and planarity, 5PGA lacked essential groups to interact with other subsite amino acid residues in all the proteases. In most cases, 5PGA atoms interacted mainly with S2 residues mainly via hydrophobic interactions. Occasionally, a hydrogen bond was observed in VP-2, KP-2 (single) and in KP-3 (double) (Table 2).

5PGA offered a good starting template to query chemical databases for structurally similar compounds. 186 compounds, analogous to 5PGA, were obtained from the ZINC database, and docked against 11 proteins. The results showed that about fifty of the ZINC compounds had very high binding affinities against *plasmodial* proteases while indicating marginal selectivity on human cathepsins (Supplementary Fig. S2). To facilitate the identification of potential hits against the *plasmodial* homologs, an energy cut off of −7.0 kcal mol^−1^ was implemented. Five compounds with good predicted inhibitory profiles (broad activity spectrum, low docking energies against *plasmodial* proteases and selectivity against human cathepsins) were selected for MD simulations and binding free energy (BFE) calculations (Figs 1 and 3, Table 2). These compounds are ZINC36371307, ZINC03869631, ZINC04532950, ZINC04579000 and ZINC052477224. As with 5PGA, the ZINC hits had a fused heterocyclic ring structure with an additional carbon chain of varied length making them highly nonpolar. The additional chain interacted with other subsite residues hence the increased binding affinities as compared to 5PGA (Figs 2 and 3). For ZINC36371307, due to the short chain (R-CH(CH3)_2_), the number of hydrophobic interactions were fewer compared to the rest of the ZINC hits. However, the inherent presence of several methyl (CH3) groups resulted in increased S2 residues interacting with ZINC36371307 compared to 5PGA. For ZINC03869631, ZINC04532950, ZINC04579000 and ZINC05247724, the docking energies observed were lower due to the existence of an increased number of interactions (hydrophobic and hydrogen bonding(s)) with other subsites (Figs 3 and 5, Table 2). In most of the cases, most ligands did not interact with any residues in the S3 of all the proteases.

Evaluation of drug-likeness is an integral part of drug discovery especially at the initial stages^45^. By considering the physicochemical properties of a compound using *in silico* approaches, its molecular impact *in vivo* is mainly determined by its bioavailability and toxicity^46^. As determined by DruLito, the identified hits passed most of the Ro5 requirements with the exception of the octanol-water partition (LogP) coefficient (Fig. 2). This can be linked to the increased size of their alkyl side chains, the steric nature of their nuclei and the low number of hydrophilic substituents. However, as this serves as a guide, more drug-like derivatives may be obtained through chemical modifications.

### Molecular dynamics

**Figure 6:**
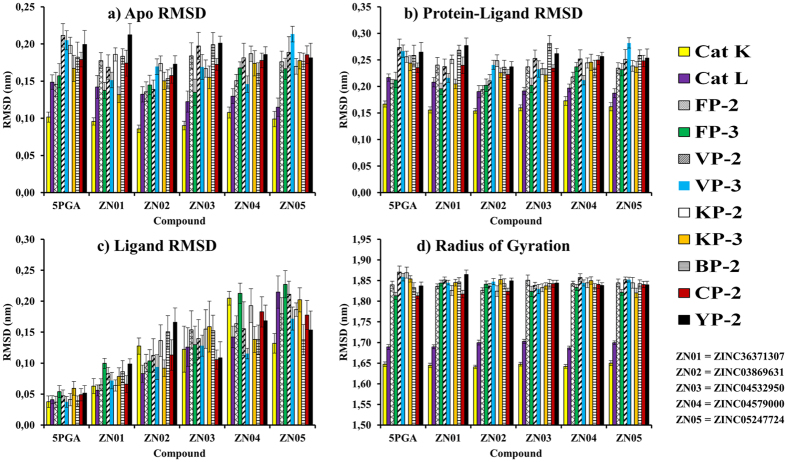
Conformational stability of the different protein complexes with 5PGA and the selected ZINC hits during the last 12 ns of MD simulations with GROMACS. The RMSD of (**a**) apo structure (**b**) holo system (**c**) ligand only and (**d**) radius of gyration. Standard deviations are shown by the error bars.

Conformational changes between each protein and selected hits were analysed through MD simulations. To determine the stability and mechanistic aspects of the protein-ligand interactions, RMSD and radius of gyration (Rg) of all protein-ligands systems were determined. The RMSD of all starting protein-ligand configurations increased during the equilibration phase and converged after 5 ns (Supplementary Fig. S3). Figure 6 shows the average RMSD of the C-alpha atoms for the apo and holo forms of all the proteases, as well as ligands during the last 12 ns of MD simulations. RMSD is commonly used to access the dynamic stability of systems as it is a global measure of protein fluctuations. The magnitude of fluctuations as depicted by the error bars indicates that all the systems attained stable conformations after equilibration. Apo forms of the human cathepsins had the lowest RMSD with that of Cat K being 0.10 ± 0.1 nm and Cat L 0.14 ± 0.1 nm. For the *plasmodial* proteases, the RMSD values were 0.18 ± 0.3 nm. To determine protein regions exhibiting higher flexibility, the RMSF per residue was calculated. Higher local fluctuations occurred in the loop regions (Supplementary Fig. S4). The largest flexibility was occurred at the characteristic inherent high fluctuating β-hairpin loop feature of the *plasmodial* proteases. The binding of ligands did not affect the proteins’ overall conformational diversity significantly, as there was no major change between the apo and holo RMSD values (Fig. 6a,b). Ligand RMSDs (Fig. 6c) were low for 5PGA and ZINC36371307 (of ~0.05 and ~0.85 nm respectively), as both compounds have planar structures and they lack of rotational bonds. In ZINC04532950, ZINC04579000, and ZINC05247724, more fluctuations were observed as the more the number of rotational bonds inherent in a ligand, the greater the fluctuations are. The Rg was calculated to determine the compactness of each protein system during the simulations. All systems were compact, with the Cat K having the lowest Rg of 1.65 nm. The human cathepsins had an average Rg of 1.8 to 1.88 nm (Fig. 6d). To further confirm the stability of the protein-ligand systems, the DSSP algorithm was used to evaluate the changes in secondary structure during MD simulations. In all systems, there were no significant changes in structural elements observed during the entire simulation time. As seen with FP-2 and Cat L in association with 5PGA, ZINC03869631 and ZINC05247724, the helical and β-sheet content remained constant during the MD simulations (Supplementary Fig. S5). This further confirmed the stability of our systems.

**Figure 7:**
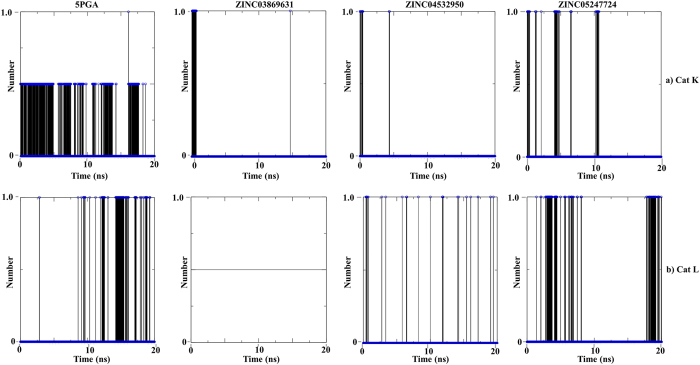
The average number of intermolecular H-bonds of (**a**) Cat k and (**b**) Cat L in complex with 5PGA, ZINC03869631, ZINC04532950 and ZINC05247724 during a 20 ns MD simulation.

**Figure 8:**
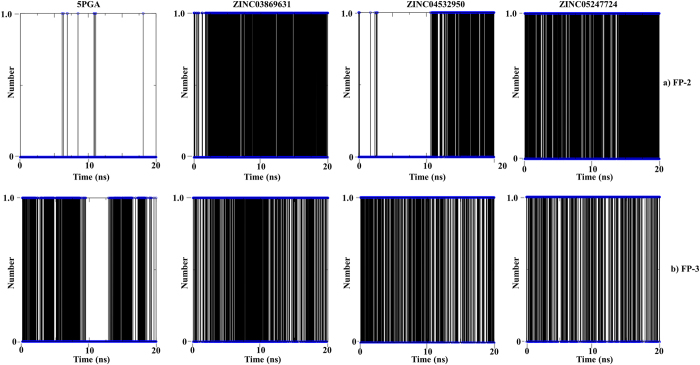
The average number of intermolecular H-bonds of (**a**) FP-2 and (**b**) FP-3 in complex with 5PGA, ZINC03869631, ZINC04532950 and ZINC05247724 during a 20 ns MD simulation.

Using the g_hbond tool in GROMACS, the number of hydrogen bonds and their occupancy during the MD simulations were determined. In most cases, the hydrogen bonds at the docking level (Table 2) were maintained during the MD simulations. For human cathepsins, the hydrogen bond occupancy between protein amino acid residues and ligand atoms was lower compared to that in *plasmodial* proteases (Figs 7 and 8). For Cat K, the hydrogen bond formed between Asn187 (catalytic domain numbering) and 5PGA was stable during the first 5 ns but fluctuated substantially during the later stages of MD simulations. At docking stage, no hydrogen bond was observed in 5PGA-Cat L complex. However, after 8 ns of MD simulations, the carbonyl oxygen in 5PGA changed orientation allowing the formation of a weak hydrogen bond with Lys118. For ZINC03869631, the H-bond with Gln143 of Cat K was on for less than 0.5 ns during equilibration while in Cat L, there was no H-bond formed during the entire simulation. For ZINC04532950 and ZINC05247724, a similar trend of unstable H-bond formation was observed. However, in *plasmodial* proteases, the hydrogen bond occupancy was higher except in FP-2 when in complex with 5PGA. The hydrogen bonds formed between FP-2 and FP-3 with ZINC03869631, ZINC04532950 and ZINC05247724 showed greater stability (Fig. 8). The observed differences in hydrogen bond formation can be of valuable use in the design of further derivatives with better binding affinities and selectivity for the *plasmodial* and human cathepsins respectively.

### Binding free energy calculations

**Figure 9:**
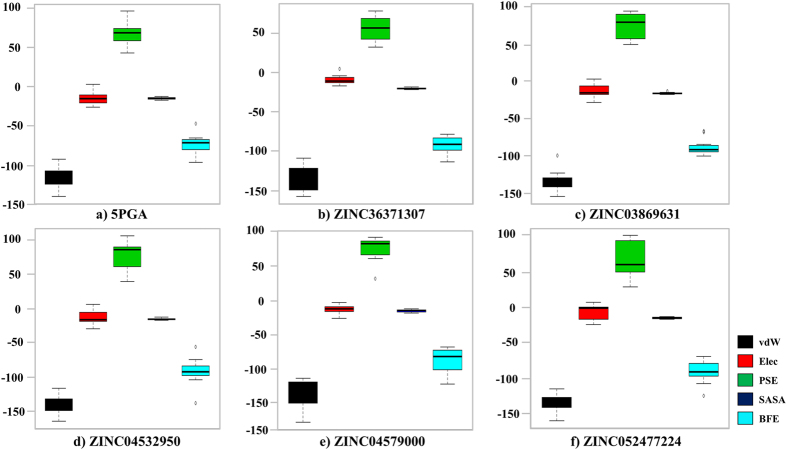
Box plots showing the distribution of the various interaction energies of different ligands.

The binding site of clan CA group of enzymes, including all the proteins studied here, consists of four defined subsites *viz.* S1, S2, S3 and S1’ (Fig. 5). Depending on the chemical nature of the ligand, different interactions ranging from hydrophobic, hydrogen, electrostatics and *pi-pi* interactions are formed between the ligand atoms and the distinct subsite residues. Each of the individual interactions contributes either positively or negatively to the overall binding free energy. In our previous work with the same protein systems^31^, polar solvation energies (PSE) impaired binding of cyanopyrimidines while van der Waals (vdW) and electrostatic forces favoured the binding process. Correspondingly, from the current results, the major impairment to binding in all systems was PSE while vdW principally favoured binding (Fig. 9). However, there was a drop in the contributions from electrostatic terms, a fact that can be explained by the few number of hydrogen bonds that were formed between the protein-ligand groups due to the chemical nature of the ligands (Figs 7 and 8). As shown in Table 3, 5PGA had the lowest binding affinities in most systems followed by ZINC36371307, just as observed in the docking studies.

### Key interactions and insights for inhibitor design

**Figure 10:**
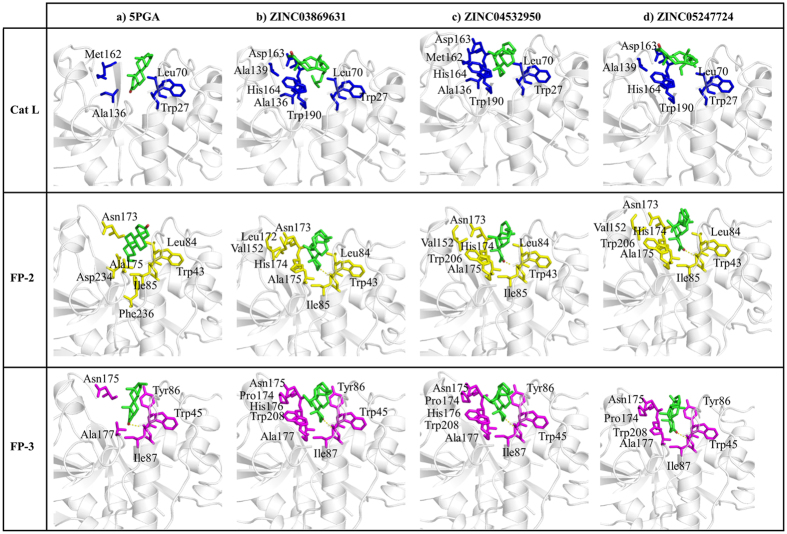
Binding pocket amino acid residue interactions patterns of bound 5PGA, ZINC03869631, ZINC04532950, and ZINC05247724 with Cat L (blue), FP-2 (yellow) and FP-3 (magenta). Hydrogen bonds are depicted by a yellow dotted line.

**Figure 11:**
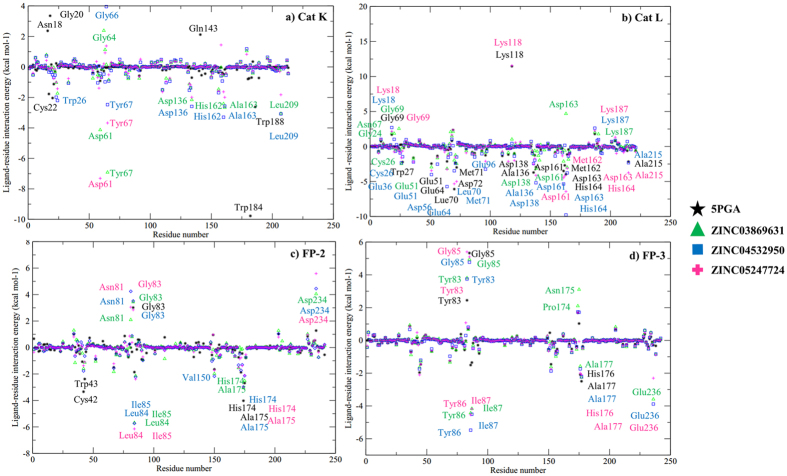
Per-residue decomposition analysis of 5PGA and the selected ZINC compounds when in complex with (**a**) Cat K, (**b**) Cat L, (**c**) FP-2 and (**d**) FP-3. Amino acids with a positive energy value impair the binding and vice versa.

From the ligand binding poses and binding free energy results, amino acid residues key to the binding process can be determined. Both 5PGA and the best ZINC hits (ZINC03869631, ZINC04532950, and ZINC05247724) exhibited different binding conformations between human Cat L and the FPs (Fig. 10). In Cat L, the ligands showed diverse binding poses in comparison with the *plasmodial* proteases. In the FPs, all the ligands consistently bound with the same pose with an exception of FP-2-5PGA. In Cat L, Trp27, Leu70, Ala136, Asp163 and His164 were the main residues involved in the binding of the ligands. In FP-2 and FP-3, Ile85 and Ile 87 participated in hydrogen bond formation with an oxygen atom present across the ligand cohort. To further determine the energetic contributions of the identified residues, a per-residue decomposition of the overall binding energies was performed. For Cat K, Asn18, Gly20 and Gln143 impaired binding while Cys22, Trp184 and Trp188 made significant contributions to the binding of 5PGA (Fig. 10a). In the case of the ZINC compounds, binding was mainly promoted by S2 and S1’ amino acid residues (Asp61, Tyr67, Asp136, His162, Ala163 and Leu209). For Cat L, Lys18, Gly69, Lys118 and Lys187 were the main residues disfavouring binding with Cys26, Glu51, Leu70, Met71, Asp138, Asp161, Met162, His164 and Ala215 favouring binding (Fig. 11). For FP-2, polar residues Asn81, Gly83 and Asp234 hindered the binding of ligands with non-polar residues Leu84, Ile85 and Ala175 favouring binding. In FP-3, polar residues Tyr83 and Gly85 disfavoured binding with Tyr86, Ile87, Ala177 and Glu236 making significant contributions to the binding of ligands. As was seen in docking studies, significant contributions to the binding of ligands (negative or positive) were by S2 and S1’ subsite residues.

## Conclusion

The central role of FPs in the pathogenesis and survival of *plasmodia* parasites in the host erythrocytes makes them attractive antimalarial drug targets. However, only a few peptide based inhibitors against these key molecular structures exist^47^. Besides the poor pharmacological profiles of these peptide inhibitors, their proneness to degradation by host proteases makes them unsuitable to be used as drugs. Our work set out to discover non-peptide compounds with predictive activity not only against FP-2 and FP-3 but also their homologs from other significant *Plasmodium* species. The use of SBVS approach with MD simulations and BFE calculations allowed us to identify an initial SA compound, 5PGA, and five further potential hit compounds from the ZINC database with a promising inhibitory activity against FP-2, FP-3 and their *plasmodial* homologs. As determined by docking studies, additional chemical groups are necessary in 5PGA to increase its potency, especially if they are designed to interact with key residues within the S1’ subsite. Our previous work with cyanopyrimidines^31^ indicated that S1’ subsite residues can provide further selectivity for *plasmodial* proteins as opposed to human homologs, beside S2 subsite interactions. Also, 5PGA lacks hydrogen bond forming atoms besides the terminal oxygen group. Although hydrophobicity feature of a ligand is very important to pass through the parasite membrane, the ligand should also have hydrogens for solubility reasons.

Three out of the top five hits obtained from ZINC database showed very promising inhibitory profiles. Although these hits had an extra carbon tail, docking studies revealed that they were not interacting with S1’ residues either. All the identified hits possessed most drug-like properties with an exception of high hydrophobic, increased nRBs in the ZINC hits and flatness factors that are known to contribute to the high hit attrition rate^48^. Thus, some chemical modifications to address the identified undesirable properties may be necessary. Interestingly though, the hits had a cholesterol-like nucleus, and might be well tolerated by human subject to further investigation. Overall, as these compounds showed encouraging selectivity between human and *plasmodial* cysteine proteases, we believe that our findings provide a suitable starting point for further development as well as detailed *in vitro* and *in vivo* analyses.

## Additional Information

**How to cite this article**: Musyoka, T. M. *et al.* Structure Based Docking and Molecular Dynamic Studies of *Plasmodial* Cysteine Proteases against a South African Natural Compound and its Analogs. *Sci. Rep.* **6**, 23690; doi: 10.1038/srep23690 (2016).

## Supplementary Material

Supplementary Information

## Figures and Tables

**Table 1:** Position of the catalytic domain of all proteins used and the corresponding domain numbering.

Protein	Position in whole sequence	Catalytic domain numbering
FP-2	244–484	1–243
FP-3	250–492	1–242
VP-2	246–487	1–242
VP-3	253–493	1–241
KP-2	252–495	1–244
KP-3	240–479	1–240
BP-2	228–468	1–241
CP-2	231–471	1–241
YP-2	232–472	1–241
Cat-K	115–329	1–215
Cat-L	113–333	1–221

**Table 2:** A summary of interacting amino acid residues with the various ligands under study upon docking.

Protein/Cmpd	5PGA	ZINC36371307	ZINC03869631	ZINC04532950	ZINC04579000	ZINC05247724
Cat K	W184, (**N187**)	W26, Y67, A134, L160, H162, A163, L209	W26,W67, A134, A137, N161, H162, A163, W184, (**Q143**)	W26, Y67, A134, Q143, N161, H162A163, W184, (**Q143**)	Q21, W26, Y67, A134, N161, H162, A163	Q21, W26, Y67, A134, A163
Cat L	W27, L70, A136, M162	Q20, Q22, L145, H164, W190, W194	W27, L70, A136, A139, D163, H164, W190	W27, L70, A136, M162, D163, H164, W190	Q22, L145, F146, H164, W190, W194, (**Q20,H164**)	W27, L70, A139, D163, H164, W190
FP-2	W43, L84, I85, N173, A175, D234, F236	W43, L84, I85, L172, H174, A175, D234	W43, L84, I85, V152, L172, N173, H174, A175, (**I85**)	W43, L84, I85, V152, N173, H174, A175, W206, (**I85**)	N81, L84, I85, Q171, L172, N173, A175, (**I85**)	W43, L84, I85, V152, N173, H174, A175, W206 (**I85**)
FP-3	W45, Y86, N175, A177 (**I87**)	W45, Y83, Y86, I87, P174, A177	W45, Y86, I87, P174, N175, H176, A177, W208, (**I87**)	W45, Y86, I87, P174, N175, H176, A177, W208, (**I87**)	W45, Y86, I87, P174, N175, A177, W208, (**I87**)	W45, Y86, I87, P174, H176, A177, (**I87**)
VP-2	W44, Y82, F85, I86, N174, A176, E235 (**I86**)	W44, Y82, F85, I86, P173, N174, A176	W44, F85, I86, V153, P173, N174, H175, A176, W207, (**I86**)	W44, F85, I86, V153, P173, N174, H175, A176, (**I86**)	W82, F85, I86, P173, N174, A176, W207, E235, (**I86**)	W44, F85, I86, V153, P173, N174, H175, A176, W207, E235, (**I86**)
VP-3	Q36, N38, V157, W206, W210	N38, A152, V157, H174, W206, W210	W43, I85, A152, V157, N173, H174, A175, W206	W43, N84, I85, A152, V157, N173, H174, A175, W206	W43, N84, I85, P172, N173, H174, A175, Q234	Q36, N38, V157, H174, W206, K209, W210, (**H174**)
KP-2	W44, L85, I86,N174,A176 (**I86**)	W44, L85, I86, P173, N174, A176	W44, L85, I86, P173, N174, H175, A176, W207, E235, (**I86**)	W44, L85, I86, P173, N174, H175, A176, W207, E235, (**I86**)	L85, I86, P173, N174, A176, W207, E235, (**I86**)	W44, L85, I86, P173, N174, H175, A176, 207, E235, (**I86**)
KP-3	W42, F84, N148, T171, N172, A174 (**I84, N148**)	W42, F83, I84, N148, T171, N172, A174	W42, F83, I84, N148, T171, N172, H173, A174, W205, (**I84**)	W42, F83, I84, N148, V151, T171, N172, H173, A174, W205, (**I84,N148**)	W42, D80, F83, I84, N148, T171, N172, A174, (**I84**)	W42, F83, I84, N148, T171, N172, H173, A174, (**I84**)
BP-2	Q37, A41, E158, W207, W211	K39, A41, V153, E158, H175, W207, W211	Q37, A41, W44, L86, A150, V153, N174, H175, A176, W207	Q37, A41, W44, V153N174, H175, A176, W207	Q37, A41, W44, V153, E158, N174, H175, A176, W207	Q37, A41, W44, V153, N174, H175, A176, W207
CP-2	A41, W44, I85, L86, P87, 150A, N174, A176, Q234, Y236	Q37, R39, A41, Q158, H175, W207, W211	A41, W44, I85, L86, A150, F172, A173, N174, H175, A176, W207, Q234	A41, W44, I85, L86, A150, F172, A173, N174, H175, A176, W207, Q234	Q37, A41, W44, L86, A150, A173, N174, A176, W206	Q37, A41, W44, A153, Q158, H175, W207
YP-2	Q37, K39, A41, V153, W207, (**K39**)	A41, W44, I85, L86, A150, Y172, A173, N174, A176, Q234	Q37, A41, W44, I85, L86V153, Y172, N174, H175, A176, W207	A41, W44, I85, V153, Y172, A173, H175, A176, W207, Q234, (**Q234**)	Q37, A41, W44, V153, N174, H175, A176, W207	Q37, A41, W44, V153, N174, H175, A176, W207

Enclosed in brackets are residues forming H-bonds. Please note that the residue numbers are according to catalytic domains. For actual protein numbering, please see Table 1.

**Table 3:** g_mmpdbsa interaction energy and binding free energy of the various protein and ligand complexes used.

Protein	Compound					
	5PGA	ZINC36371307	ZINC03869631	ZINC04532950	ZINC04579000	ZINC05247724
Cat K	−78.6 ± 0.2	−113.0 ± 0.2	−99.2 ± 0.1	−91.8 ± 0.2	−81.3 ± 0.2	−93.4 ± 0.2
Cat L	−93.2 ± 0.2	−88.4 ± 0.1	−96.7 ± 0.2	−136.7 ± 0.2	−99.4 ± 0.2	−125.0 ± 0.2
FP-2	−86.7 ± 0.1	−81.2 ± 0.1	−86.7 ± 0.2	−83.8 ± 0.2	−70.6 ± 0.2	−80.1 ± 0.2
FP-3	−62.1 ± 0.2	−93.1 ± 0.2	−91.4 ± 0.2	−96.7 ± 0.2	−102.3 ± 0.2	−92.1 ± 0.2
VP-2	−65.3 ± 0.2	−101.7 ± 0.2	−91.3 ± 0.2	−92.3 ± 0.2	−81.6 ± 0.2	−107.9 ± 0.2
VP-3	−71.5 ± 0.1	−78.2 ± 0.2	−66.8 ± 0.2	−73.9 ± 0.2	−121.7 ± 0.7	−101.7 ± 0.2
KP-2	−68.2 ± 0.2	−109.9 ± 0.2	−91.4 ± 0.2	−81.9 ± 0.2	−68.0 ± 0.2	−71.5 ± 0.1
KP-3	−44.5 ± 0.2	−81.8 ± 0.2	−68.2 ± 0.2	−55.3 ± 0.2	−70.6 ± 0.1	−69.8 ± 0.2
BP-2	−65.6 ± 0.2	−90.1 ± 0.2	−92.7 ± 0.2	−84.0 ± 0.2	−74.2 ± 0.2	−82.7 ± 0.2
CP-2	−75.4 ± 0.1	−89.8 ± 0.2	−100.2 ± 0.2	−103.0 ± 0.2	−119.1 ± 0.3	−91.8 ± 0.2
YP-2	−62.9 ± 0.2	−76.6 ± 0.2	−84.7 ± 0.1	−96.7 ± 0.2	−86.4 ± 0.2	−79.3 ± 0.1
