# Supplementary material for: Structure Based Docking and Molecular Dynamic Studies of Plasmodial Cysteine Proteases against a South African Natural Compound and its Analogs
Source: Sci Rep. 2016 Mar 31;6:23690. doi: 10.1038/srep23690 (PMC4814779; doi:10.1038/srep23690)
Supplement: Supplementary Information [file srep23690-s1.pdf]

# Structure Based Docking and Molecular Dynamic Studies of *Plasmodial* Cysteine Proteases against a South African Natural Compound and its Analogs

Thommas M. Musyoka[1], Aquillah M. Kanzi[1,2], Kevin A. Lobb[3] & Özlem Tastan Bishop[1]*

[1]Research Unit in Bioinformatics (RUBi), Department of Biochemistry and Microbiology, Rhodes University, Grahamstown, South Africa

[2]Department of Genetics, Forestry and Agricultural Biotechnology Institute (FABI), Faculty of Natural and Agricultural Sciences, University of Pretoria, Pretoria, South Africa

[3]Department of Chemistry, Rhodes University, Grahamstown, South Africa

Correspondence and requests for materials should be addressed to Ö.T.B. (email: O.TastanBishop@ru.ac.za)

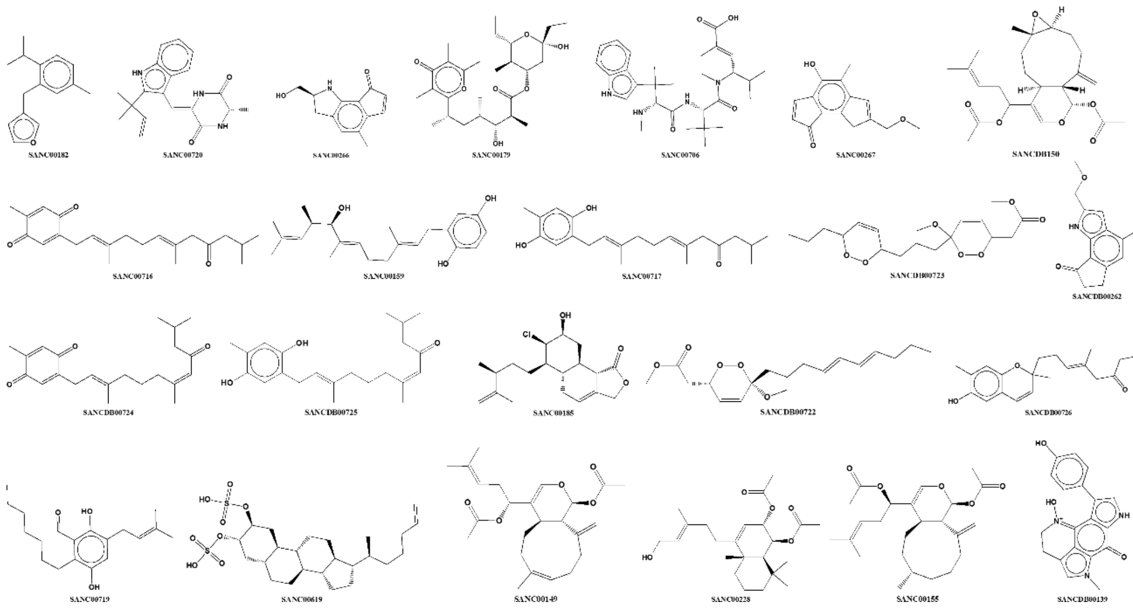


**Supplementary Figure S1.** 2D of South African non-peptidic compounds with SANCDB identity numbers used for initial docking studies.

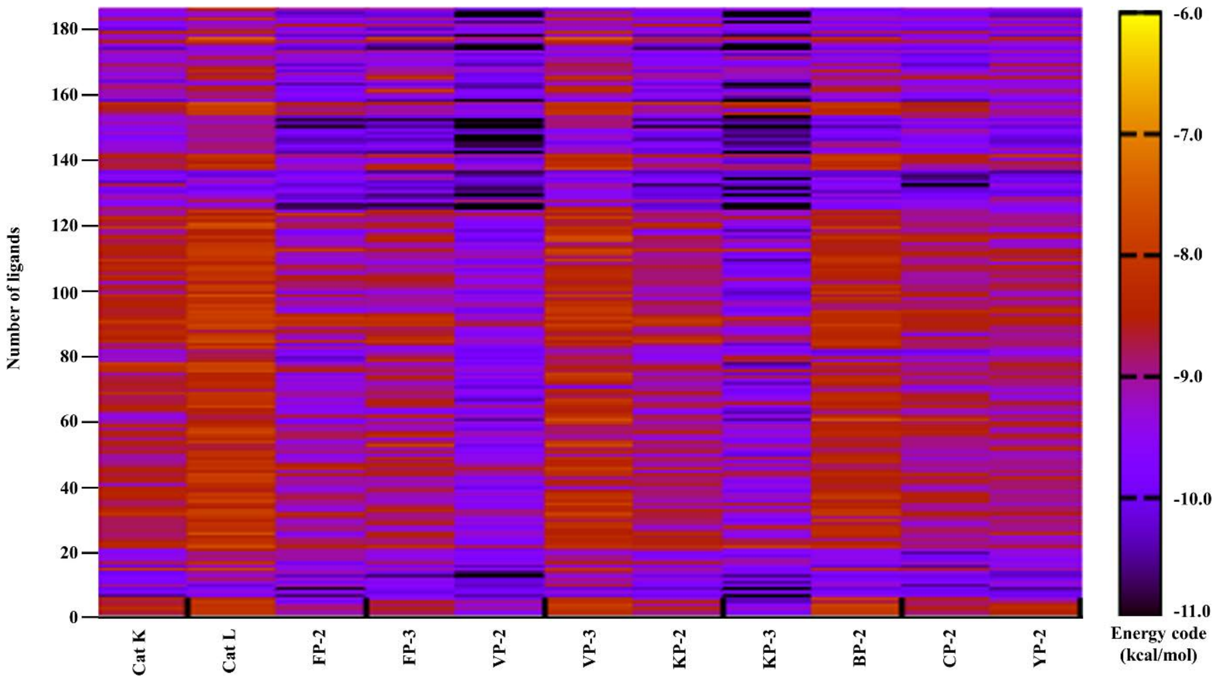


**Supplementary Figure S2.** Docking results on all proteases using AutoDock4.2 for all 186 ZINC hits obtained via 3D structure similarity search using 5PGA as the query template.

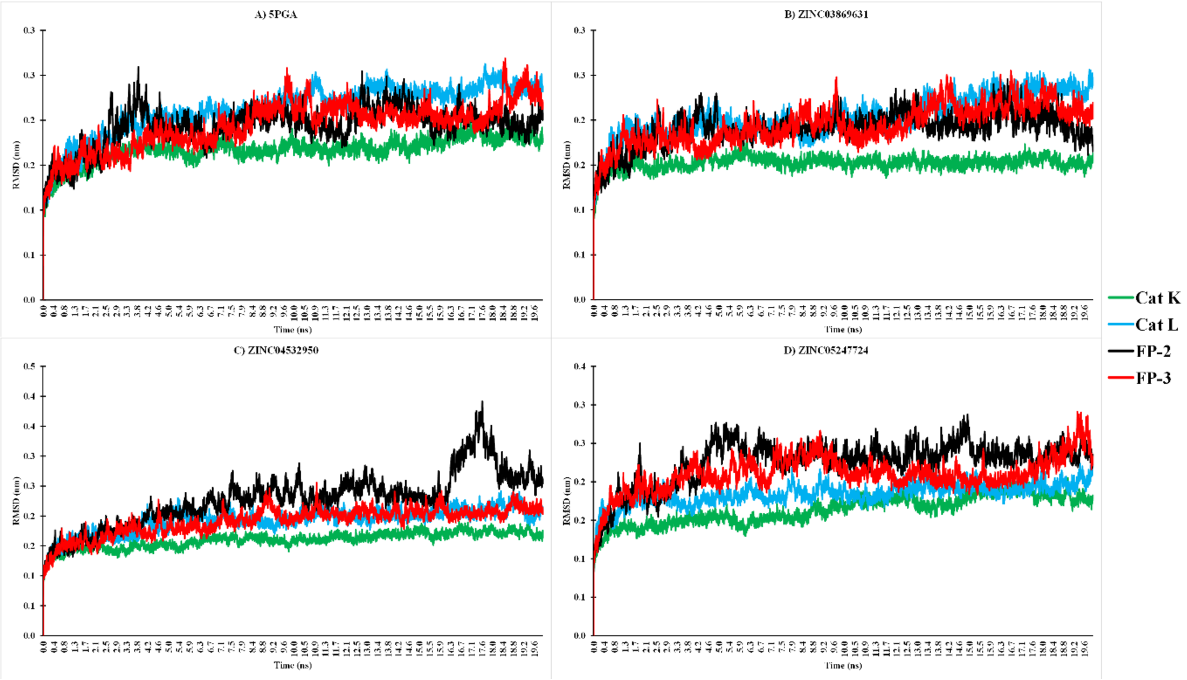


**Supplementary Figure S3:** RMSD plots of FP-2, FP-3 and Cat L in complex with **a)** 5PGA, **b)** ZINC03869631, **c)** ZINC04532950, and **d)** ZINC05247724.

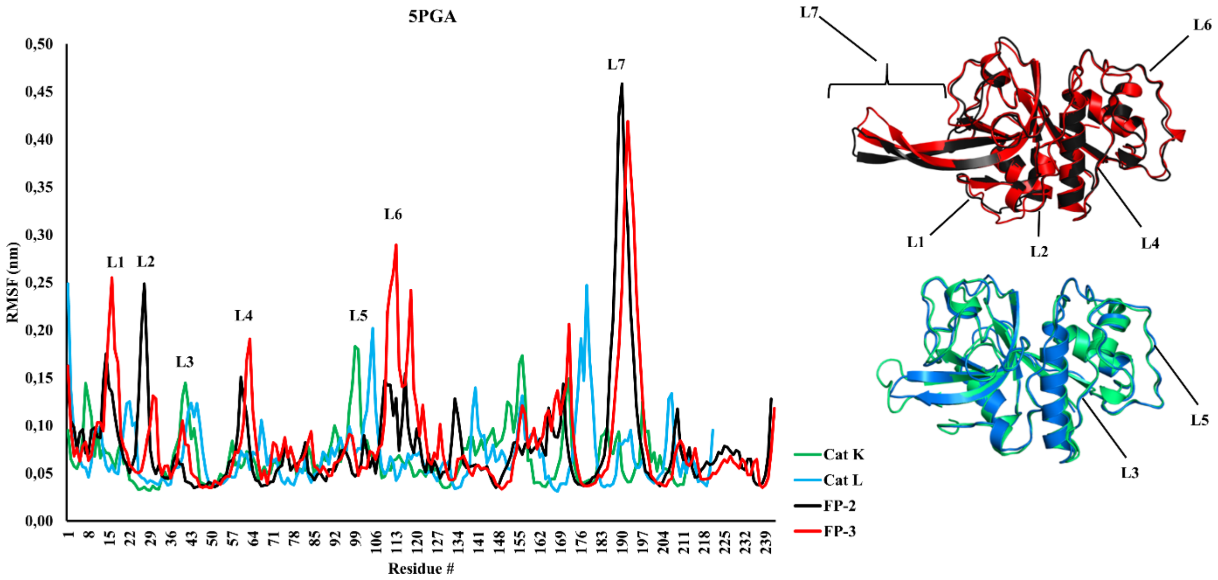


**Supplementary Figure S4:** RMSF plot of FP-2, FP-3 and Cat L in complex with 5PGA. The cartoon representation shows the different loop regions (LX) that exhibit highest fluctuations.

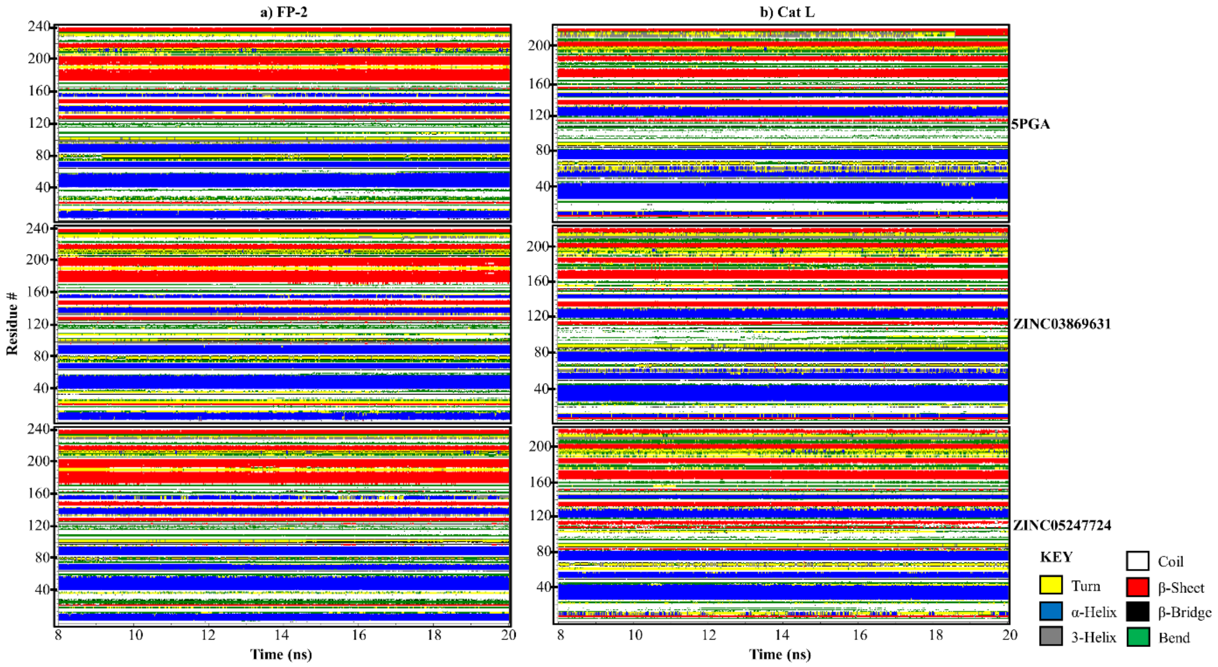


**Supplementary Figure S5.** Conformational evolution of secondary structure elements of a) FP-2 and b) Cat L in complex with 5PGA, ZINC03869631 and ZINC05247724 during the last 12 ns of MD simulation as determined by do_dssp of GROMACS 4.6.5.
